# Survey of Microstructures and Dimensional Accuracy of Various Microlattice Designs Using Additively Manufactured 718 Superalloy

**DOI:** 10.3390/ma17174334

**Published:** 2024-09-01

**Authors:** Huan Li, Benjamin Stegman, Chao Shen, Shiyu Zhou, Anyu Shang, Yang Chen, Emiliano Joseph Flores, R. Edwin García, Xinghang Zhang, Haiyan Wang

**Affiliations:** 1 School of Materials Engineering, Purdue University, West Lafayette, IN 47907, USA; li4868@purdue.edu (H.L.); bstegma@purdue.edu (B.S.); shen569@purdue.edu (C.S.); zhou1351@purdue.edu (S.Z.); shanga@purdue.edu (A.S.); chen4919@purdue.edu (Y.C.); flore170@purdue.edu (E.J.F.); redwing@purdue.edu (R.E.G.); 2 School of Electrical and Computer Engineering, Purdue University, West Lafayette, IN 47907, USA

**Keywords:** additive manufacturing, microlattices, crack formation, architecture, inconel 718, transmission electron microscopy (TEM)

## Abstract

Microlattices hold significant potential for developing lightweight structures for the aeronautics and astronautics industries. Laser Powder Bed Fusion (LPBF) is an attractive method for producing these structures due to its capacity for achieving high-resolution, intricately designed architectures. However, defects, such as cracks, in the as-printed alloys degrade mechanical properties, particularly tensile strength, and thereby limit their applications. This study examines the effects of microlattice architecture and relative density on crack formation in the as-printed 718 superalloy. Complex microlattice design and higher relative density are more prone to large-scale crack formation. The mechanisms behind these phenomena are discussed. This study reveals that microlattice type and relative density are crucial factors in defect formation in LPBF metallic alloys. The transmission electron microscopy observations show roughly round γ″ precipitates with an average size of 10 nm in the as-printed 718 without heat treatment. This work demonstrates the feasibility of the additive manufacturing of complex microlattices using 718 superalloys towards architectured lightweight structures.

## 1. Introduction

Significant advancements in additive manufacturing (AM) technology have been made in building components with complex geometry and manufacturing complex materials with advanced mechanical properties [1,2,3,4,5,6]. Metallic microlattices, a subset of complex geometrical parts, have been successfully manufactured using the AM technique. These microlattices consist of repeated 2D/3D patterns that create continuous, low-density structures, making them essential for lightweight applications in the aeronautics and astronautics industries, where reducing weight is both cost-effective and advantageous [7,8]. Moreover, microlattices have demonstrated significant enhancement in energy absorption at high strain rates compared to traditional bulk materials. These microlattices have also seen applications in medical implants for modulus adjustments, improving bone acceptance through stress shielding, and in creating auxetic structures with a negative Poisson’s ratio [9,10,11]. Various AM technologies have been adopted for fabricating microlattices, including aerosol jet printing, stereolithography variants, inkjet writing, investment casting, and laser powder bed fusion (LPBF) [12,13,14,15,16]. Each technique has its own trade-offs, such as material compatibility, geometric resolution, and size limitations. LPBF stands out due to its high-resolution capabilities, fine laser spot size, and minimal need for post-build processing.

Various types of microlattices have been constructed using LPBF. Common repetitive microlattice structures include cubic, body-centered cubic (bcc), honeycomb, diamond, and gyroid structures, among others [10]. The struts, which link the repeating subsets, possess numerous characteristics, such as height, diameter, and curvature, all of which influence the mechanical performance and deformation mechanisms of microlattices [17]. Given the complexity of these microlattice designs and various types of defects that may arise during AM [10], it is crucial to validate the dimensional accuracy and structural integrity of AM microlattices. This validation is essential for optimizing mechanical properties by adjusting the lattice’s design, strut and node geometries, relative density, and the composition of alloys [18]. Huynh et al. successfully printed an IN 718 bcc microlattice using LPBF and studied its fatigue behavior [19]. Under compression testing at room temperature, its yield strength is 238 MPa, and the fracture strain is 4.6%. During compression fatigue testing, when the maximum fatigue stress is two-thirds of the yield strength (159 MPa), its fatigue life is ~5000 cycles. Overall, both the compression and fatigue performance are relatively poor. Moreover, Banait et al. studied the effective hybridization of face-centered cubic (fcc) and octet in AM IN 718 microlattices [20] and proved that the efficiency of lattice hybridization is highly dependent on the microstructure of the base materials and on the size of the reinforcement lattice domains. Hazeli et al. printed IN 718 microlattices of octet, dodecahedron, and diamond structures using LPBF technology [21]. The trend in maximum porosity formation in the node center and minimum porosity at the beginning of the node was observed for all three microlattices. The microlattices of a 718 nickel-based alloy produced through LPBF has been increasingly investigated, largely due to its broad application in the aerospace industries [22]. This superalloy features a face-centered cubic (fcc) γ matrix reinforced by γ′ and γ″ phases, allowing it to withstand high temperatures up to 650 °C [23]. The as-printed 718 Ni-based superalloy exhibits a columnar grain morphology oriented along the printing direction (Z direction) and a more equiaxed grain microstructure in the XY plane. The rapid solidification rates and dramatic thermal gradients inherent in the LPBF process led to the formation of dislocation cell boundaries, different from the conventional dendritic structures in casted 718 superalloys [24]. This process causes regional solute segregation of high-melting-point elements, leading to the accumulation of Nb and Mo near the cell walls [25,26]. The dislocation pile-up serves as a pathway for solute diffusion [27]. Remnant heat during the LPBF technique can cause the precipitation of ultra-fine Laves and delta phases at the intersections of the cell walls in as-printed 718 alloys [25,26]. The presence of Laves and delta phases is undesirable due to their limited slip systems, which contribute to brittle fractures [28].

Defects, such as cracks, can significantly degrade the tensile properties of AM alloys and microlattices [29]. For example, Stegman et al. revealed that BCC microlattices containing microcracks display a low tensile strength of only 20 MPa and a strain of 20%, while BCC microlattices with minimal cracks exhibit a high tensile strength of 80 MPa and a strain of 44% [30,31]. In this research, we explore the effects of the designs and relative densities of microlattices on dimensional accuracy and defect formation in the LPBF 718 superalloy. The analyses of defects in microlattices reveal new findings on the differences in crack formation mechanisms for various 718 microlattices.

## 2. Materials and Methods

**Figure 1:**
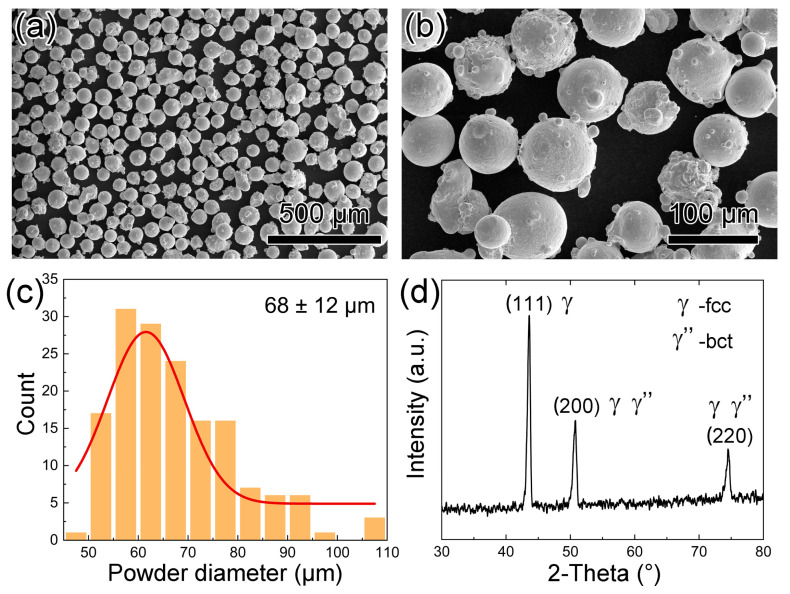
(**a**,**b**) Low- and high-magnification SEM micrographs of 718 superalloy powders. (**c**) The histogram and Gaussian fit curve of powder diameters. (**d**) XRD pattern of the 718 superalloy powder.

The 718 superalloy powder from Praxair Surface Technologies (Indianapolis, IN, USA) was produced through traditional gas atomization methods, and the composition is detailed in Table 1. The powder particles are uniformly spherical and of high quality, as depicted in Figure 1a,b. The particle size distribution is 68 ± 12 μm, as shown in Figure 1c. Additionally, the XRD analysis of the 718 superalloy powder reveals the presence of γ-fcc and γ″-bct phases, as shown in Figure 1d.

**Figure 2:**
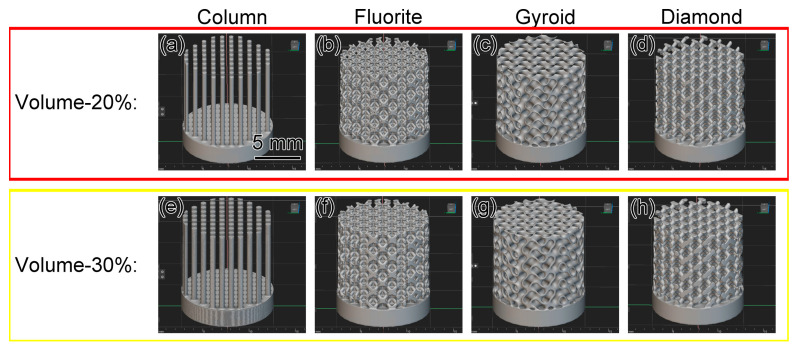
Computer-designed simulation images of various microlattices: (**a**) Column-20%, (**b**) Fluorite-20%, (**c**) Gyroid-20%, (**d**) Diamond-20%, (**e**) Column-30%, (**f**) Fluorite-30%, (**g**) Gyroid-30%, and (**h**) Diamond-30%.

nTopology software (https://www.ntop.com/) was used to create multiple microlattice designs (Figure 2). Four microlattice structures—column, fluorite, gyroid, and diamond—were designed, each with relative densities of 20% and 30%. Eight types of microlattices were designed, named Column-20%, Column-30%, Fluorite-20%, Fluorite-30%, Gyroid-20%, Gyroid-30%, Diamond-20%, and Diamond-30%. These microlattices, each with a diameter and height of 9.8 mm, were connected by a thin base. The relative density of each sample was set by adjusting the thickness of this thin-walled connection, as outlined in Table 2.

Six specimens were printed for each design to ensure experimental repeatability. These specimens were fabricated via LPBF using the 718 superalloy powder on a 316L stainless steel build plate, with an SLM 125 LPBF instrument (Nikon SLM Solutions, Long Beach, CA, USA) with an oxygen concentration of 0.1% (1000 ppm). Our previous work identified the printing parameters that produce the highest density components, namely a laser power of 285 W, a scan speed of 960 mm/s, a hatch spacing of 100 µm, and a layer thickness of 40 µm [31]. A laser spot size of 70 µm and a build plate temperature of 80 °C were used. A wire electrical discharge machine (EDM) instrument was used to remove specimens from the build plate. X-ray diffraction (XRD) 2-theta peak analysis was conducted using a PANalytical Empyrean X’pert PRO MRD diffractometer (Malvern Panalytical, Malvern, UK) with a 2 × Ge (220) hybrid monochromator to select for Cu Kα1 radiation at a rotation speed of 20°/min. Scanning electron microscopy (SEM) micrographs were captured using a Thermo Fisher Teneo microscope. The microlattices were characterized using SEM at room temperature, and the dimension (such as thickness) of microlattices was measured 15 times from SEM images to obtain the average thickness and standard deviation. The TEM sample was fabricated in a dual beam SEM-FIB (Thermo Scientific Helios G4 UX Dual Beam, Waltham, MA, USA). The microstructure was then characterized using a 200 kV transmission electron microscope (TEM, Thermo Fisher Talos 200X TEM/STEM with ChemiSTEM technology, X-FEG, and superX EDS, Waltham, MA, USA).

## 3. Results

**Figure 3:**
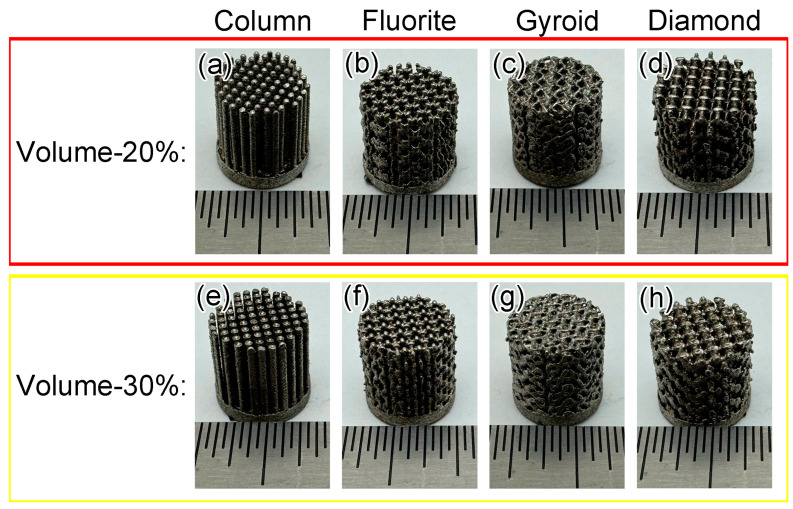
Optical images of printed actual microlattices: (**a**) Column-20%, (**b**) Fluorite-20%, (**c**) Gyroid-20%, (**d**) Diamond-20%, (**e**) Column-30%, (**f**) Fluorite-30%, (**g**) Gyroid-30%, and (**h**) Diamond-30%. The minimum scale in this Figure represents 0.8 mm.

Figure 3 shows optical images of printed cylindrical microlattices with a diameter and height of 9.8 mm. All specimens were printed successfully based on the original design, and displayed a metallic luster without visible cracks.

**Figure 4:**
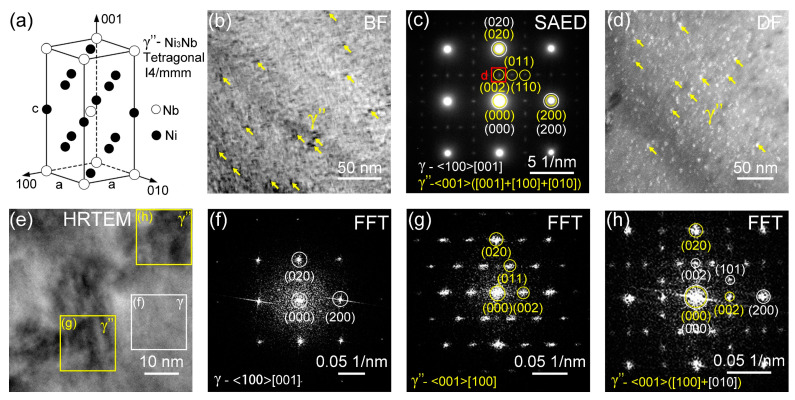
TEM micrographs of Column-20% microlattice. (**a**) The schematic of the bct (DO22) γ″-Ni_3_Nb. (**b**) BF-TEM image showing the γ matrix and the γ″-Ni_3_Nb nanoprecipitates. (**c**) The SAED patten showing the presence of γ and γ″ phases. (**d**) DF-TEM image using the superlattice spot (d) in Figure 4c showing high-density γ″ nanoprecipitates. (**e**) HRTEM image of the γ grain with several γ″ precipitates. (**f**) FFT of <100>[001]-oriented γ matrix. (g) and (h) FFT of <001>[100]-oriented γ″ and <001>([100]+[010])-oriented γ″ at location (**g**) and (**h**) in Figure 4e, separately.

Figure 4 displays TEM micrographs of the Column-20% specimen. The schematic of the bct (DO22) γ″-Ni3Nb is shown in Figure 4a. Figure 4b is a low-magnification bright field (BF) TEM image displaying precipitates within γ. The corresponding SAED patterns in Figure 4c shows the presence of γ″ in three orientations: <001> ([001]+[100]+[010]). Figure 4d is a dark field (DF) TEM image by using the superlattice spots from (002) γ″. The DF TEM image proves the presence of γ″ and their uniform distribution in the matrix. Moreover, the morphology of γ″ precipitates is circular. A high-resolution TEM (HRTEM) image (Figure 4e) and the corresponding fast Fourier transformation (FFT) of the γ matrix (Figure 4f) precipitates in Figure 4g,h confirm the presence of γ″ precipitates oriented along the <001> ([100]+[010]) orientations.

**Figure 5:**
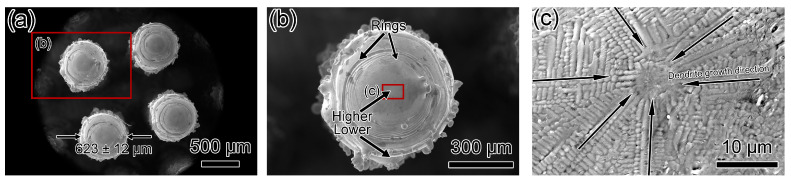
SEM micrographs of top-down view of Column-20% microlattice. (**a**,**b**) Low-magnification micrographs showing circular columns with ring patterns. The center of the post is higher than the edges. (**c**) SEM micrograph showing dendrite growth along the radial direction towards the center.

**Figure 6:**
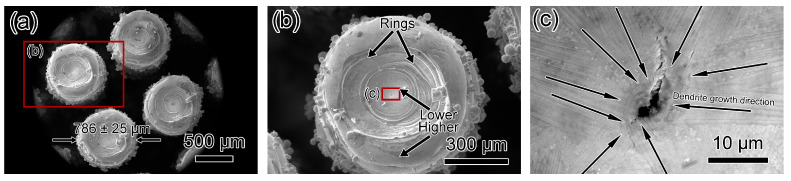
SEM micrographs of top-down view of Column-30% microlattice. (**a**,**b**) Low-magnification micrographs showing circular columns. The center of the post is lower than the peripherals. (**c**) SEM micrograph showing dendrite growth along the radial direction towards the center. Small pores ~10 μm and minor cracks were observed in the center.

Figure 5 and Figure 6 present SEM micrographs of Column-20% and Column-30% microlattices. Both types consist of small columns with wall thicknesses of 623 ± 12 μm and 786 ± 25 μm, respectively (Figure 5a and Figure 6a). Dimensional deviations for Column-20% and Column-30% are 23.1% and 26.6%, respectively, compared to the designed thicknesses of 506 μm and 621 μm (Table 2). This observation indicates the minimal impact of relative density on dimensional deviation. Figure 5b and Figure 6b show individual columns, both with rings and no large-scale surface cracks. However, the center of columns in the Column-20% specimen is higher than its edges, while the Column-30% specimen’s center is lower. The surfaces are not smooth due to rings, and the surfaces of the columns have residual partially sintered powders. Figure 5c reveals dendritic crystals without a preferred orientation for the Column-20% specimen, whereas Figure 6c shows that Column-30% has dendritic columns towards the center containing a 17 μm long crack, and the dendrites stopped growing at this small-scale crack.

**Figure 7:**
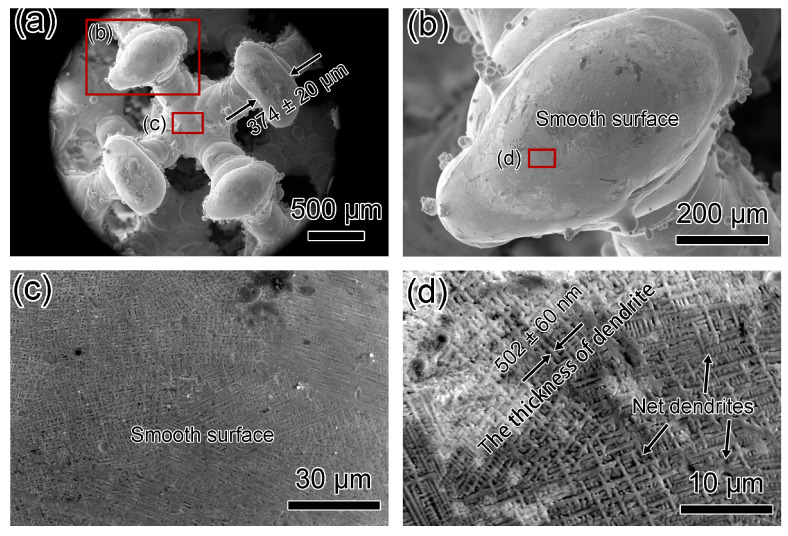
SEM micrographs of top-down view of Fluorite-20% microlattice. (**a**,**b**) Low-magnification micrographs showing fluorite structure. (**c**,**d**) SEM micrograph showing dendrite crystals with a thickness of 502 ± 60 nm on the surface. No cracks were observed.

**Figure 8:**
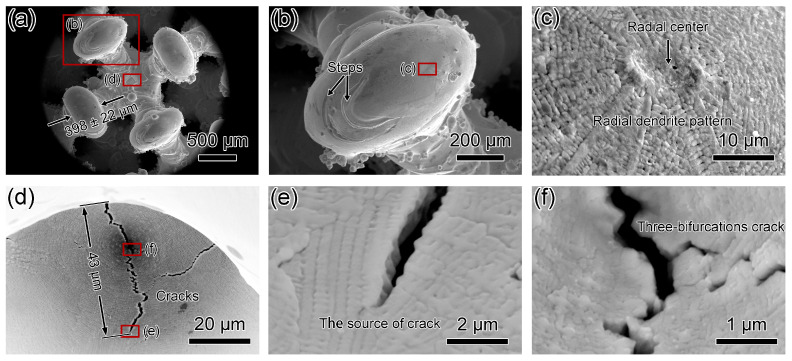
SEM micrographs showing top-down view of Fluorite-30% microlattice. (**a**,**b**) Low-magnification micrographs showing fluorite structure. (**c**) SEM micrograph showing radial dendrite pattern on the surface. (**d**) SEM micrograph showing large cracks with length of 43 μm. (**e**) SEM micrographs showing the source of the crack. (**f**) SEM micrographs showing the three-way bifurcation cracks.

Figure 7 and Figure 8 display SEM micrographs of Fluorite-20% and Fluorite-30% microlattices. Wall thicknesses for Fluorite-20% and Fluorite-30% are 374 ± 20 μm and 398 ± 22 μm, respectively (Figure 7a and Figure 8a), compared to the n-topology designed thicknesses of 305 μm and 387 μm (Table 2). Dimensional deviations for Fluorite-20% and Fluorite-30% are 22.6% and 2.9%, respectively, indicating a higher relative density of results in lower dimensional deviations and higher accuracies. Figure 7b and Figure 8b show nodes with centers higher than the edges. The Fluorite-20% node surface is smooth, while the Fluorite-30% node surface has scattered steps and partially sintered powder. Both specimens consist of dendritic crystals with net and radial morphologies, respectively (Figure 7c and Figure 8c). Figure 7c and Figure 8d show the centers of two microlattices connecting four nodes. The center of Fluorite-20% appears smooth and crack-free, while Fluorite-30% has large-scale cracks, the longest being 43 μm. Figure 8e,f details intergranular cracks, including some three-way crack segments.

**Figure 9:**
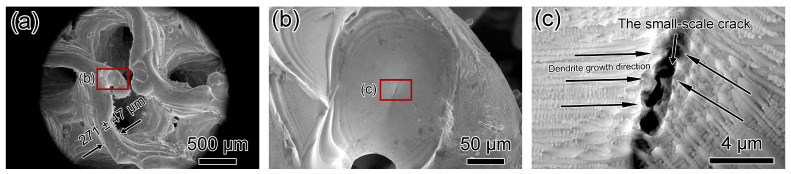
SEM micrographs of top-down view of Gyroid-20% microlattice. (**a**,**b**) Low-magnification micrographs showing gyroid structure. (**b**) SEM micrograph showing one of the nodes. (**c**) SEM micrograph showing dendrite growth direction and a small crack on the surface.

**Figure 10:**
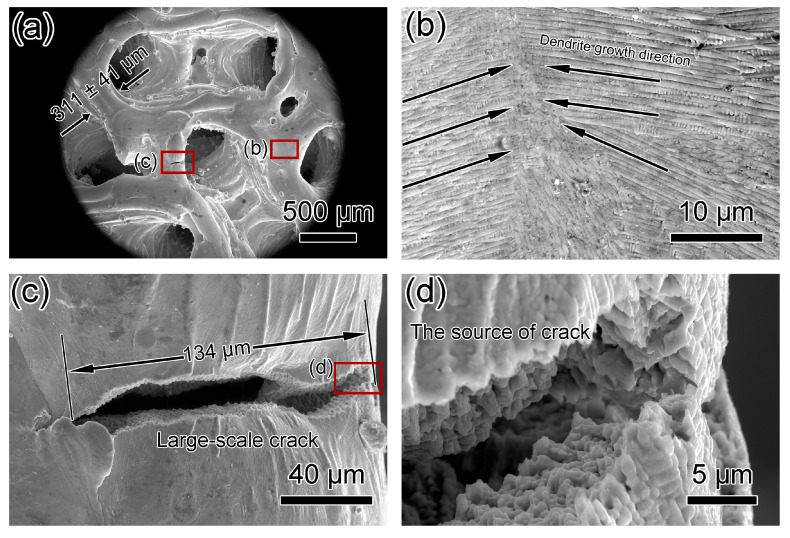
SEM micrographs of top-down view of Gyroid-30% microlattice. (**a**) Low-magnification micrograph showing gyroid structure. (**b**) SEM micrograph showing dendrite crystals and dendrite growth direction on the surface. (**c**) SEM micrograph showing a large-scale crack with a length of more than 100 μm on the surface. (**d**) High-magnification micrograph showing the source of the crack.

Figure 9 and Figure 10 show SEM micrographs of Gyroid-20% and Gyroid-30% microlattices. Wall thicknesses for Gyroid-20% and Gyroid-30% are 271 ± 47 μm and 311 ± 41 μm, respectively (Figure 9a and Figure 10a). Dimensional deviations for Gyroid-20% and Gyroid-30% are 30.5% and 0.4%, respectively, compared to the designed thicknesses of 208 and 310 μm (Table 2), indicating higher relative density results in smaller deviation and higher dimensional accuracy. Among all of the microlattices printed in this study, Gyroid-20% has the thinnest wall thickness (271 ± 47 μm) and Gyroid-30% has the lowest dimensional deviation (0.4%). Both specimens show directional dendritic growth (Figure 9c and Figure 10b). A small crack, 8 μm in length, was observed in Gyroid-20%, compared to a 134 μm long crack in the Gyroid-30% microlattice (Figure 10c,d). 

**Figure 11:**
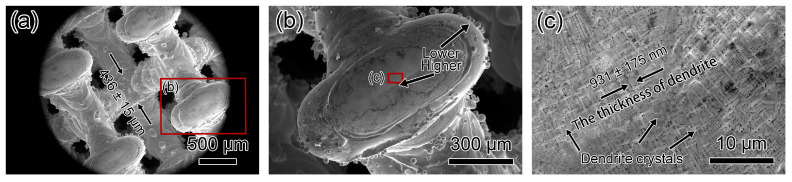
SEM micrographs of top-down view of Diamond-20% microlattice. (**a**,**b**) Low magnification micrographs showing the diamond’s structure, and the node has higher centers than edges. (**c**) SEM micrograph showing randomly oriented dendrite crystals on the surface. No cracks were observed.

**Figure 12:**
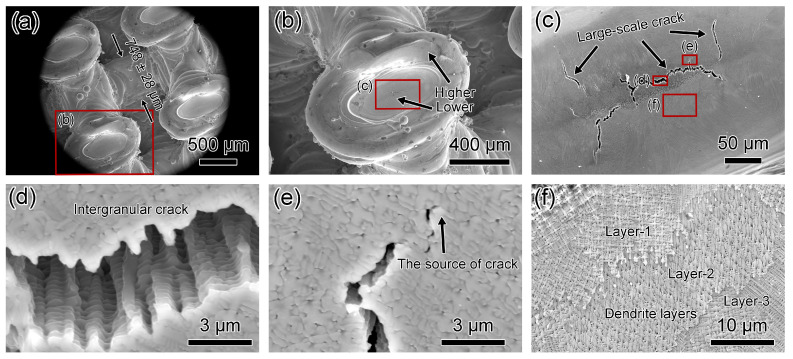
SEM micrographs of top-down view of Diamond-30% microlattice. (**a**,**b**) Low magnification micrographs showing the diamond’s structure, and the node centers are lower than edges. (**c**) SEM micrograph showing several large-scale cracks. (**d**) SEM micrograph showing an inter dendritic crack. (**e**) SEM micrograph capturing the crack tip. (**f**) SEM micrograph showing the layers of dendrite crystals.

Figure 11 and Figure 12 depict SEM micrographs of Diamond-20% and Diamond-30% microlattices. Wall thicknesses for Diamond-20% and Diamond-30% are 436 ± 15 μm and 748 ± 28 μm, respectively (Figure 11a and Figure 12a). Dimensional deviations for Diamond-20% and Diamond-30% are 1.5% and 37.2%, respectively, compared to the original designed thicknesses of 430 and 545 μm (Table 2), indicating that a smaller relative density leads to less deviation. Figure 11b shows a typical node for the Diamond-20% microlattice with centers higher than edges. In contrast, Figure 12b shows the opposite for the Diamond-30% microlattice, with the node centers being lower than the edges. As shown in Figure 11c, the Diamond-20% has randomly oriented dendritic crystals with an average primary dendrite width of 931 ± 175 nm. No cracks were observed from the surface of Diamond-20% microlattices. In comparison, numerous cracks are present at the node centers for the Diamond-30% specimen (Figure 12c). The crack length can reach 210 μm. The cracks propagate along dendritic boundaries, with sharp crack tips similar to Fluorite-30% (Figure 12d,e). The Diamond-30% specimen also shows a layered morphology of dendritic crystals (Figure 12f).

**Figure 13:**
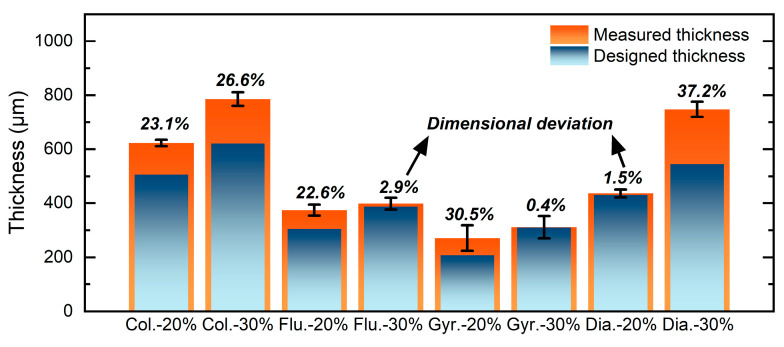
Histogram of measured thickness and designed thickness with the dimensional deviation value.

Measured thickness, designed thickness, deviation ratio, and cracks in microlattices are summarized in Table 2, which is also plotted in Figure 13.

## 4. Discussion

Prior studies on LPBF 718 superalloys have mostly focused on simple structures, like cubes or cylinders [32,33]. The primary advantage of LPBF technology is its ability to print complex structures such as Octet, Honeycomb, and BCC lattices due to its excellent dimensional accuracy. There are limited studies on the microstructures of 718 microlattices [33]. Our study shows various successfully printed microlattices, including column, fluorite, gyroid, and diamond microlattices with 20% and 30% relative density, using LPBF technology. SEM studies show that the design and relative density of microlattices may change the crack formation behavior.

### 4.1. The Formation of γ″ Precipitates in the As-Printed 718 Alloy without Heat Treatment

The bct (DO_22_) γ″-Ni_3_Nb precipitate plays a significant role in strengthening the 718 superalloys [19,20,21,34,35,36,37]. For instance, these precipitates increase the yield strength and creep resistance at elevated temperatures [19,20,21]. In general, γ″ precipitates mostly form after heat treatment [20]. It is likely that γ″ precipitates formed as a consequence of indirect heat treatment during the melting of overlayers and nearby laser tracks [34,35]. The studies by Cao et al. on heat-treated LPBF IN 718 revealed that γ″ precipitates have a platelet-shape with dimensions of 10–50 nm [37]. However, in our study, the γ″ precipitates are ~10 nm with a spherical shape. The SAED pattern in Figure 4c and FFTs (Figure 4f–h) show the orientation relationship between γ and γ″: <001>([001]+[100]+[010])_γ″_//(100)[001]_γ_, in agreement with prior studies [34,35,36,37]. 

### 4.2. Dimensional Accuracy of Microlattices Using LPBF 

The high spatial resolution of LPBF has been demonstrated in this study. In general, LPBF has advantages to print fine microstructures compared to other AM techniques, such as direct energy deposition and e-beam melting. Prior studies have shown that the smallest feature size achievable in LPBF is typically on the order of several hundred microns [38,39,40,41,42]. Here, we show the success in achieving the small wall thickness of 270 μm for the Gyroid-20% microlattice compared to all other microlattices we printed using LPBF. Such a small dimension with little cracking is difficult to achieve in general, even for the LPBF technique. The laser spot size of 70 µm used in the current study helped to achieve the small wall thickness in 718 Ni alloys. 

The dimensional accuracy varies from 1 to 37% in this study. Wu et al. [39] reported that the dimensional deviations of 30 Inconel 718 samples fabricated using LPBF varied from 1% to 44%. When the designed thickness was 138 μm, the dimensional deviation reached as large as 44%. Moreover, Noronha et al. [42] fabricated 286 Ti-6Al-V samples using LPBF with dimensional deviations from 45% to 66%. Therefore, the dimensional deviations (1–37%) in our research are with a typical range (1–37%) based on prior studies. The general trend analyzed from an array of microlattices printed in this study suggests that the dimensional accuracy may be limited by the complexity of the design and spatial resolution of the LPBF technique. Printing tiny dimensional structures (thin walls) challenges the dimensional accuracy. Adding complexity to the design also aggravates geometric deviation from the original design. However, further exploration and optimization of printing strategies can potentially improve the dimensional accuracy. 

### 4.3. The Influence of Microlattice Architectures on Crack Formation

Among several microlattices studied here, the column microlattices were mostly free of large-scale cracks. Complex microlattices like diamond, fluorite, and gyroid have intricate geometries with interconnections. During the LPBF process, rapid heating and cooling cycles create significant thermal gradients within these microlattices [43]. This uneven heat distribution and localized high cooling rates result in high residual stresses that can exceed the yield strength of 718 superalloys, leading to cracking [44]. In contrast, simpler microlattices like columns have more uniform geometries, allowing for even heat distribution and consistent cooling rates. This uniformity results in lower residual stresses and reduces the likelihood of crack formation [45]. The intricate geometries of diamond, fluorite, and gyroid structures also cause stress concentrations at sharp corners, thin sections, and high-curvature locations, which serve as initial sites for crack nucleation. Conversely, the uniform cross-sectional area and simple shape of column structures lead to a more homogeneous distribution of residual stresses, reducing stress concentrations and preventing crack formation [30]. Nickel-based superalloys like IN 718 tend to solidify with a dendritic microstructure. In complex geometries, varying cooling rates cause non-uniform dendrite growth and increased segregation of alloying elements at grain boundaries, making these areas more vulnerable to crack formation [33]. In the simpler column structures, cooling rates are more uniform, resulting in consistent microstructural features and fewer defects [46].

### 4.4. The Influence of Relative Density on Crack Formation

Prior studies show that the relative density of microlattices is an important parameter that influences the yield strength and specific strength [32]. Samples with lower relative density can have a greater yield strength and consume less materials [47]. We found that microlattices with higher relative density were more prone to crack formation [43]. Microlattices with higher relative density have more interior links (struts), which restrict contraction and increase strain and residual stresses, leading to cracks [44]. The rapid heating and cooling rates of LPBF technology also contribute to this aggravated cracking behavior, as higher relative density increases the amount of 718 alloy being melted and solidified, creating significant thermal gradients and resulting in cracks [45]. Additionally, a higher relative density promotes extensive dendritic growth in the IN 718 alloys, making them more susceptible to cracking [31]. The increasing heat per unit volume in microlattices with greater relative density can lead to localized overheating, causing thermal stress and cracking. During solidification, solutes like Nb, Mo, and Ti can segregate along grain boundaries, and higher relative density can enhance this segregation, weakening grain boundaries and increasing grain boundary susceptibility to cracking [46]. Reducing the relative density to 20% appears to largely suppress cracking in the diamond and fluorite microlattices. 

Beyond studying defect formation in microlattices, future research should explore the effects of different architectures on mechanical properties [33]. For example, Stegman et al. found that the tensile behaviors of IN 718 differ prominently among straight bar, honeycomb, and BCC microlattices, with honeycomb microlattices displaying a high work-hardening rate and substantial grain reorientation [30]. Their study suggests that the architecture of microlattices profoundly affects the mechanical behavior and deformation mechanisms in 718 superalloys. Future studies will focus on comparing the mechanical properties of these microlattices through tensile and compression tests to elucidate their deformation mechanisms [48,49,50,51,52,53,54,55,56].

## 5. Conclusions

This study compares the dimensional accuracy and defect formation in four sets of microlattice designs, i.e., Column, Fluorite, Gyroid, and Diamond, with relative densities of 20% and 30%. All eight microlattices were successfully printed by LPBF without catastrophic fractures. SEM studies show that all of the microlattice designs have certain dimensional deviations from their original design within a range of 1–37%. The column microlattices with different relative density were free of large-scale cracks, while fluorite, gyroid, and diamond microlattices displayed moderate microcracks. This difference indicates that complex structures tend to aggravate crack formation due to heterogeneous heat distribution and that local high cooling rates induce residual stresses. Additionally, microlattices with higher relative density were more prone to microscale cracks due to increasing interconnected struts, which may elevate residual stresses. TEM studies show that nanoscale round γ″ precipitates with an average size of 10 nm present in the as-printed 718 without heat treatment, and the orientation relationship between γ″ and γ is determined to be (001)([001]+[100]+[010])_γ″_//(100)[001]_γ_. The demonstration of AM-718 superalloy microlattices with versatile designs presents a promising path towards architectured light weight structures and components.

## Figures and Tables

**Table 1:** Alloy powder chemistry for the 718 superalloy powder.

	Ni	Cr	Fe	Nb	Mo	Ti	Al	Mn	Si	Co	C	Ta	O	N	B
718 Powder (wt%)	Bal.	18.71	18.12	5.12	3.04	0.93	0.49	0.35	0.15	0.09	0.08	0.05	0.011	0.012	0.001

**Table 2:** Summary of measured thickness, designed thickness, deviation ratio, and cracks in microlattices. Note that the nominal density is also provided for each microlattice.

	Measured Thickness (μm)	Designed Thickness (μm)	Dimensional Deviation (%)	Large Cracks
Column-20%	623 ± 12	506	23.1	No
Column-30%	786 ± 25	621	26.6	No
Fluorite-20%	374 ± 20	305	22.6	No
Fluorite-30%	398 ± 22	387	2.9	Yes
Gyroid-20%	271 ± 47	208	30.5	No
Gyroid-30%	311 ± 41	310	0.4	Yes
Diamond-20%	436 ± 15	430	1.5	No
Diamond-30%	748 ± 28	545	37.2	Yes

## Data Availability

The original contributions presented in the study are included in the article; further inquiries can be directed to the Xinghang Zhang (xzhang98@purdue.edu) and Haiyan Wang (hwang00@purdue.edu).
